# Supplementary material for: Bacillus mycoides PM35 Reinforces Photosynthetic Efficiency, Antioxidant Defense, Expression of Stress-Responsive Genes, and Ameliorates the Effects of Salinity Stress in Maize
Source: Life (Basel). 2022 Jan 30;12(2):219. doi: 10.3390/life12020219 (PMC8875943; doi:10.3390/life12020219)
Supplement: Supplementary file 1 [file life-12-00219-s001.zip › life-1489246-supplementary.pdf]

Supplementary T1. Experimental design for pot studies

Treatments	Description
T0	Control
T1	300 mM NaCl
T2	600 mM NaCl
T3	900 mM NaCl
T4	*Bacillus mycoides* PM35
T5	*B. mycoides* PM35 + 300 mM
T6	*B. mycoides* PM35 + 600 mM
T7	*B. mycoides* PM35 + 900 mM

Supplementary T2. Physio-chemical properties of soil

**Soil parameters**	**Soil 1 (Pre-sowing)**	**Soil 2 (Post-harvesting)**
**Soil texture**	**Loamy**	**Loamy**
**pH**	**7.94**	**7.87**
**Electrical conductivity (dS/m)**	**1.53**	**4.49**
**Organic matter (%)**	**3.49**	**1.88**
**Available Phosphorus (mg/kg)**	**45.62**	**33.85**
**Available Potassium (mg/kg)**	**601**	**124**
**Saturation (%)**	**44**	**43**
